# Clinicopathological features of PD-L1 protein expression, EBV positivity, and MSI status in patients with advanced gastric and esophagogastric junction adenocarcinoma in Japan

**DOI:** 10.1080/15384047.2022.2038002

**Published:** 2022-02-27

**Authors:** Tsutomu Yoshida, Go Ogura, Mikiko Tanabe, Takuo Hayashi, Chiho Ohbayashi, Mizutomo Azuma, Chikara Kunisaki, Yoichi Akazawa, Soji Ozawa, Sohei Matsumoto, Takayoshi Suzuki, Akira Mitoro, Tetsu Fukunaga, Akiko Shimizu, Go Fujimoto, Takashi Yao

**Affiliations:** a Department of Pathology, Kitasato University School of Medicine, Sagamihara, Japan; b Department of Pathology, Tokai University School of Medicine, Isehara, Japan; c Division of Diagnostic Pathology, Yokohama City University Medical Center, Yokohama, Japan; d Department of Diagnostic Pathology, Main Hospital, Juntendo University, Tokyo, Japan; e Department of Diagnostic Pathology, Nara Medical University, Kashihara, Japan; f Department of Gastroenterology, Kitasato University School of Medicine, Sagamihara, Japan; g Department of Surgery, Gastroenterological Center, Yokohama City University Medical Center, Yokohama, Japan; h Department of Gastroenterology, School of Medicine, Juntendo University, Tokyo, Japan; i Department of Gastroenterological Surgery, Tokai University School of Medicine, Isehara, Japan; j Department of Surgery, Nara Medical University, Kashihara, Japan; k Division of Gastroenterology and Hepatology, Tokai University School of Medicine, Tokai University, Isehara, Japan; l Department of Gastroenterology, Nara Medical University, Kashihara, Japan; m Department of Gastroenterology and Minimally Invasive Surgery, School of Medicine, Juntendo University, Tokyo, Japan; n Oncology Medical Affairs, MSD K.K., Tokyo, Japan; o Medical Affairs, MSD K.K., Tokyo, Japan; p Department of Human Pathology, Juntendo University Graduate School of Medicine, Tokyo, Japan

**Keywords:** Adenocarcinoma, Japan, microsatellite instability, PD-L1, stomach

## Abstract

This real-world study examined the prevalence of programmed death ligand-1 (PD-L1) expression and assessed the frequency of microsatellite instability-high (MSI-H) status and Epstein-Barr virus (EBV) positivity in Japanese patients with advanced gastric and gastroesophageal junction (GEJ) adenocarcinoma. This multicenter (5 sites), retrospective, observational study (November 2018–March 2019) evaluated Japanese patients with advanced gastric and GEJ adenocarcinoma after surgical resection (Stage II/III at initial diagnosis) or unresectable advanced cancer (Stage IV). The primary objectives were prevalence of PD-L1 expression (combined positive score [CPS] ≥1), MSI status, and EBV positivity. Tumor specimens of 389/391 patients were analyzed (male, 67.1%; mean age, 67.6 ± 12.2 years); 241/389 (62%) were PD-L1 positive, 24/379 (6.3%) had MSI-H tumors, and 13/389 (3.3%) were EBV positive. PD-L1 expression was higher in tumor-infiltrating immune cells than in tumor cells for lower CPS cutoffs. Among patients with MSI-H tumors and EBV-positive tumors, 19/24 (79.2%) and 9/13 (69.2%), respectively, were PD-L1 positive. A greater proportion of patients with MSI-H tumors (83.3% [20/24]) were PD-L1 positive than those with MSI-low/stable tumors (60.8% [216/355]; *p* = .0297); similarly, an association was observed between history of *H pylori* infection and PD-L1 expression. A higher proportion of patients with MSI-H tumors demonstrated PD-L1 expression with a CPS ≥10 (66.7% [16/24]) vs those with MSI-low/stable tumors (24.8% [88/355]; *p* < .0001). The prevalence of PD-L1 positivity among Japanese patients was comparable to that in previous pembrolizumab clinical trials and studies in gastric cancer. Particularly, higher PD-L1 expression was observed in MSI-H tumors.

## Introduction

Gastric cancer (GC) is the fifth most common cancer and accounts for 5.6% of all new cancers reported worldwide in 2020.^1^

The prevalence of GC in Japan is high and despite diagnosis of GC at an early stage due to regular screening programs, the prevalence of advanced GC and mortality continue to be high. Consequently, surgical options are limited and there exists an unmet need for the treatment of advanced-stage GC.^2,3^ Therefore, numerous therapeutic options based on molecular characteristics of the tumor are being evaluated to improve GC treatment outcomes.

The Cancer Genome Atlas (TCGA) project proposed a molecular classification based on comprehensive genomic modeling, dividing GC into 4 subtypes: Epstein-Barr virus (EBV)–positive tumors, microsatellite instability-high (MSI-H) tumors, genomically stable tumors, and tumors with chromosomal instability.^4^ Among them, the correlation between high expression of programmed death ligand-1 (PD-L1) and prognosis in patients with GC has been extensively investigated. Studies report that PD-L1 expression commonly examined in tumor cells (TCs) is mostly associated with a poor prognosis and shorter overall survival (OS) in patients in East Asia,^5–7^ while a good prognosis was also reported.^8,9^ However, based on the TCGA classification, an increasing role of EBV-positive and MSI-H subtypes of GC is being observed with PD-L1 expression,^10–12^ indicating the involvement of the immune microenvironment in the development of GC.^8,13^ It was reported that PD-L1 expression in immune cells in EBV-positive or MSI-H tumors was associated with favorable prognosis.^14,15^ However, outcomes based on evaluation of these molecular characteristics are likely to be confounded by choice of assays, scoring methods used, and the stage of tumor. Currently, the combined positive score (CPS) is validated as a sensitive method to score PD-L1 expression in various cancers, including GC,^16–19^ especially near the low cutoff point.^17,18,20,21^

For example, with reference to the application of CPS as a diagnostic method, a unique anti–PD-1 inhibitor such as pembrolizumab was approved for the treatment of head and neck squamous cell cancer, esophageal squamous cell carcinoma, and triple-negative breast cancer in Japan.^22,23^ Pembrolizumab is also indicated for the treatment of adult patients with unresectable or metastatic MSI-H or mismatch repair–deficient solid tumors that have progressed following prior treatment and who have no satisfactory alternative treatment options.^24^ In Japan, pembrolizumab was approved as monotherapy for MSI-H tumors, including GC/gastroesophageal junction (GEJ) adenocarcinoma, in 2018.^25^

To date, real-world data on PD-L1 expression determined by CPS have not been evaluated in a Japanese population with advanced GC or GEJ cancer. Consequently, this real-world study examined the prevalence of PD-L1 expression (CPS ≥1) determined using a Food and Drug Administration–approved kit, MSI-H frequency, and EBV positivity in Japanese patients with advanced GC and GEJ adenocarcinoma. The primary objectives of this study were to examine the prevalence of PD-L1 protein expression (CPS ≥1) and assess MSI status and EBV positivity in Japanese patients with advanced gastric and GEJ adenocarcinoma. Other objectives were to characterize the association between PD-L1 expression and biomarker subtypes; determine the association between PD-L1 expression and patients’ clinicopathological features, including site of occurrence, stage of GC, and histological subtype; and summarize PD-L1 protein expression by CPS distribution.

## Methods

### Patients and study design

**Figure 1.**
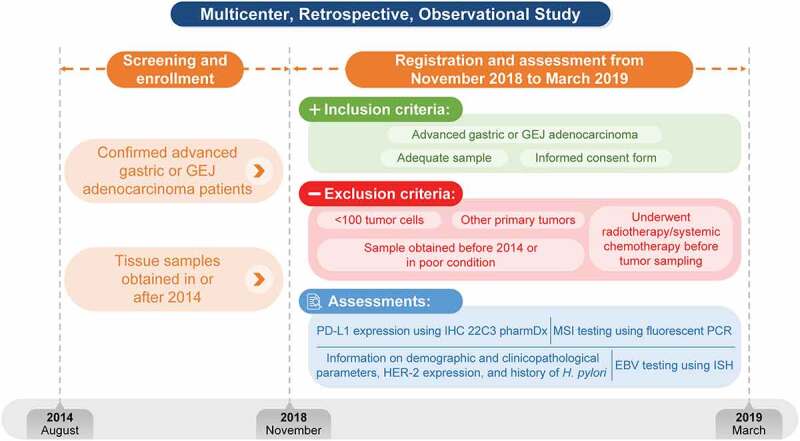
Study design EBV, Epstein-Barr virus; GEJ, gastroesophageal junction; HER-2, human epidermal growth factor receptor-2; *H. pylori, Helicobacter pylori*; IHC, immunohistochemistry; ISH, insitu hybridization; MSI, microsatellite instability; PCR, polymerase chain reaction; PD-L1, programmed death ligand-1.

This was a multicenter (5 sites in Japan: Kitasato University School of Medicine, Tokai University School of Medicine, Yokohama City University Medical Center, Juntendo University, and Nara Medical University), retrospective, observational study conducted between November 2018 and March 2019 to obtain real-world data from Japanese patients with advanced gastric and GEJ adenocarcinoma (Figure 1).

The study enrolled Japanese patients aged ≥20 years (at sampling) with Stage II or III advanced gastric and GEJ adenocarcinoma following surgical resection in or after 2014 and who experienced recurrence at or after 6 months following postoperative adjuvant chemotherapy or who were diagnosed with unresectable advanced cancer (Stage IV) and underwent tumor sampling in or after 2014. Patients were required to have archived tissue specimens adequate to provide 14 paraffin sections. All patients provided informed consent for this study. Patients were excluded if they had other primary tumor types, had undergone radiotherapy and/or systemic chemotherapy prior to tumor sampling, had specimens that had been obtained before 2014, had tissue specimens that had been sectioned >6 months prior to sampling, had <100 TCs, or had tissue specimens that had been preserved poorly. The screening method for registration in this study was to trace back and check the diagnosis date of advanced GC or metastatic GC in medical records of each patient candidate, and among these candidates, patients eligible for this study were registered in order from the newest diagnostic date in each center.

A total of 391 archived tissue specimens from Japanese patients with gastric and GEJ adenocarcinoma who have experienced recurrence or metastasis were tested for PD-L1 protein expression, MSI status, and EBV positivity.

### Assessments

PD-L1 expression was assessed using the PD-L1 IHC 22C3 pharmDx kit (Agilent Technologies, Carpinteria, CA, USA), a qualitative immunohistochemistry (IHC) assay using monoclonal mouse anti–PD-L1 clone 22C3, and measured with the EnVision FLEX visualization system on the Autostainer Link 48 (Agilent Technologies, Santa Clara, CA, USA). PD-L1 expression was determined using CPS, which was calculated as the total number of cells stained positive for PD-L1 (TCs, lymphocytes, macrophages) × 100/the total number of viable cells,^16^ and was approved in Japan as a companion diagnostic (CDx; for head and neck cancer and expanded use for esophageal squamous cell carcinoma) project. To minimize bias during evaluation of PD-L1 expression, participating pathologists were trained on scoring before the study was initiated. A CPS ≥1 indicates positive PD-L1 expression.^16^

MSI status was evaluated using a fluorescent polymerase chain reaction (PCR)-based assay. The MSI-IVD kit was developed by FALCO (Kyoto, Japan) to assess 5 mononucleotide repeat markers (BAT-25, BAT-26, NR-21, NR-24, and MONO-27) and 2 pentanucleotide repeat markers (Penta C and Penta D). The PCR products were separated by capillary electrophoresis using the Applied Biosystems PRISM® 310 or 3100 or Applied Biosystems™ 3130 or 3130xl Genetic Analyzer (Foster City, CA, USA). The output data were analyzed using the GeneMapper® software (Applied Biosystems, Foster City, CA, USA). EBV testing was performed by in situ hybridization (ISH) at a central laboratory, with the exception of 1 site where the standard protocol was used in-house.

Medical records and test results were used for the collection of demographic information, including Eastern Cooperative Oncology Group performance status (ECOG PS), history of gastrectomy, metastatic location, and number of metastatic sites; clinicopathological data (date of GC diagnosis, site of occurrence [stomach or GEJ], stage of GC based on the Union for International Cancer Control [UICC]/American Joint Committee on Cancer [AJCC] classification 7th edition, histological type and degree of differentiation, metastatic organ, and type of tumor tissue specimen); human epidermal growth factor receptor-2 (HER-2) status obtained from IHC and/or fluorescent in situ hybridization (FISH); and history of *Helicobacter pylori* (*H. pylori*) infection. The date of *H. pylori* testing was recorded on the electronic data capture (EDC), and 106 cases tested for *H. pylori* infection were included. A positive test result was considered as having a history of infection. As the testing methods had not been specified, tests such as biopsy, serum test, or breath antibody test, which are covered by insurance in Japan, were identified and included.

### Statistical analysis

Descriptive analyses were conducted to examine the prevalence of PD-L1 expression and assess MSI status and EBV positivity. Fisher’s exact test was used to differentiate the proportion of patients with PD-L1 expression by other biomarkers. Based on the planned sample size of 400 patients and the results of cohort 1 from the KEYNOTE-059 trial,^26,27^ which reported PD-L1 protein expression in approximately 57% of patients (CPS ≥1), the results of this study were expected to have a 95% confidence interval (CI) of 52.0%–61.9% for PD-L1 protein expression (CPS ≥1).

## Results

### Sample and study population

Eligible patients were registered for this study from November 2018 to March 2019. Tumor specimens from 391 Japanese patients with advanced gastric and GEJ adenocarcinoma were obtained. However, tumor specimens from 2 patients were excluded from the study because of violation of inclusion (recurrence during postoperative adjuvant chemotherapy) and exclusion (insufficient [<100] TCs) criteria; therefore, tumor specimens from 389 patients were included in the analysis set.

The majority of patients were male (67.1%), with a mean age at diagnosis of 67.6 ± 12.2 years, and most had ECOG PS in the range of 0 (47%) to 1 (26%). The most commonly occurring cancer site was the stomach (94.9%), and 17.2% of patients had a history of gastrectomy. Overall, 93.6% of patients had metastases; the majority of patients (62.7%) had metastasis in the lymph nodes, followed by the peritoneum (38.3%) and liver (30.3%). Most tumor specimens were obtained by biopsy (81.2%), followed by surgical resection (18.8%). The majority of the cancers were poorly differentiated (62.0%), and the most common cancer was of the diffuse type (48.8%). Overall, 50% (53/106) of the tumor specimens of patients tested positive for *H. pylori*. The prevalence of *H. pylori* in patients with GC was 50% (53/106). The proportion of men with vs without a history of *H. pylori* infection was 57% (30/53) vs 66% (35/53), respectively. The proportion of patients with vs without a history of *H. pylori* infection and having stomach as the tumor site was 98% (52/53) vs 89% (49/53), respectively. When characterized using the Lauren classification, the proportion of patients both with vs without a history of *H. pylori* infection and diffuse-type tumors was the highest (55% [29/53] vs 62% [33/53]), followed by intestinal-type (43% [23/53] vs 32% [17/53]) and mixed-type (2% [1/53] vs 6% [3/53]) tumors.

### Prevalence of PD-L1, MSI status, and EBV positivity

**Figure 2.**
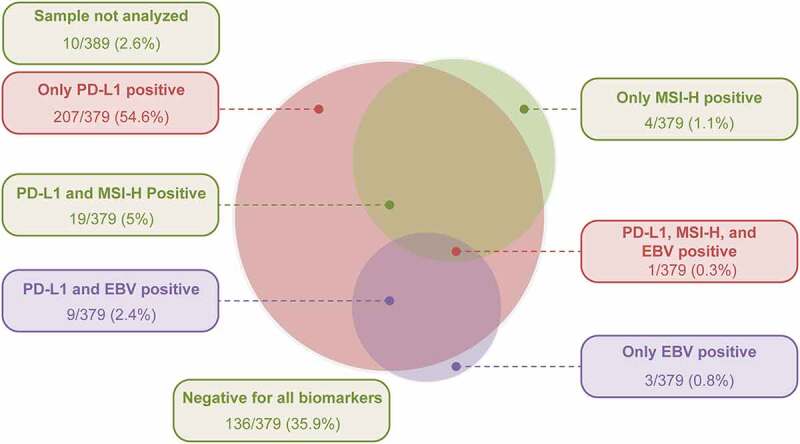
Relationship of PD-L1 expression with MSI-H and EBV-positive tumors EBV, Epstein-Barr virus; MSI-H, microsatellite instability-high; PD-L1, programmed death ligand-1.

Tumor specimens from 241/389 (62%) patients were positive for PD-L1 expression, with a CPS ≥1. Tumor specimens from 24/379 (6.3%) and 13/389 (3.3%) patients were MSI-H and EBV-positive tumors, respectively (Table 1). Tumors that were positive only for PD-L1 were detected in 207/379 (54.6%) patients (Figure 2), and tumor specimens from 136/379 (35.9%) patients were negative for all biomarkers. Among patients with MSI-H tumors and EBV-positive tumors, 19/24 (79.2%) and 9/13 (69.2%), respectively, were PD-L1 positive (Table 1 and Figure 2).

**Table 1.** Prevalence of PD-L1, MSI, and EBV expression

Biomarker	Total patients tested, *N*	Status	Patients, *n* (%)	95% CI^a^
PD-L1 expression^b^	389	Positive	241 (62.0)	56.9, 66.8
MSI status^c^	379	MSI-H	24 (6.3)	4.1, 9.3
EBV status	389	Positive	13 (3.3)	1.8, 5.6

^a^Clopper-Pearson method

^b^PD-L1 expression is positive when the CPS is ≥1 and negative when the CPS is <1

^c^Proportion of patients, including reference values

CI, confidence interval; CPS, combined positive score; EBV, Epstein-Barr virus; MSI-H, microsatellite instability-high; PD-L1, programmed death ligand-1

### Prevalence of HER-2– and PD-L1–positive tumors based on the Lauren classification

**Figure 3.**
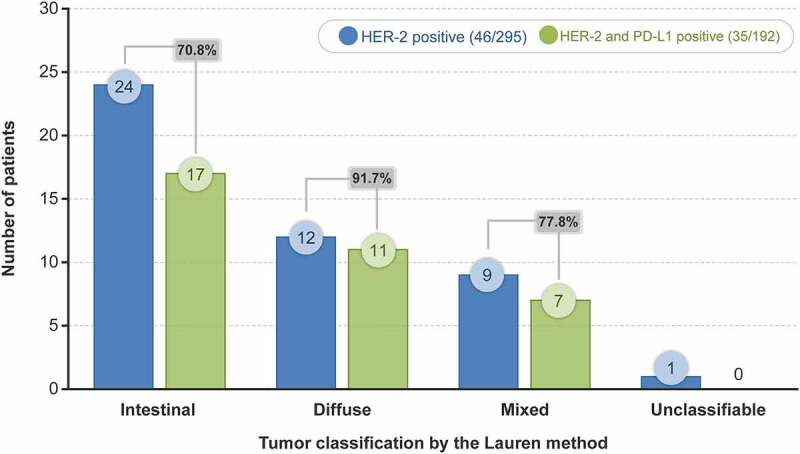
Prevalence of HER-2– and PD-L1–positive tumors based on the Lauren classification

Among 46 patients with HER-2–positive tumors, 35 (76.1%) had PD-L1–positive tumors (Table 2). A majority of the patients with HER-2–positive tumors had the intestinal type (24/46 [52.17%]), followed by diffuse type (12/46 [26.09%]), mixed type (9/46 [19.57%]), and unclassifiable type (1/46 [2.17%]). However, the proportion of patients with PD-L1–positive tumors among the HER-2–positive patients was highest for the diffuse type (11/12 [91.7%]), followed by mixed type (7/9 [77.8%]) and intestinal type (17/24 [70.8%]) (Figure 3).

**Table 2.** Association of PD-L1 expression with biomarker subtypes

Biomarker	Biomarker status	Patients with available biomarker data, *N*	PD-L1–positive patients, *n* (%)	95% CI^a^	*p*-value^b^
MSI	MSI-H	24	20 (83.3)	62.6, 95.3	.0297
	MSI-low/stable	355	216 (60.8)	55.6, 66.0	
EBV	Positive	13	10 (76.9)	46.2, 95.0	.3853
	Negative	376	231 (61.4)	56.3, 66.4	
HER-2^c^	Positive	46	35 (76.1)	61.2, 87.4	.0950
	Negative	249	157 (63.1)	56.7, 69.1	
*H. pylori*	Yes	53	44 (83.0)	70.2, 91.9	.0030
	No	53	29 (54.7)	40.4, 68.4	

^a^Clopper-Pearson method

^b^Fisher’s exact test

^c^The result is regarded as positive if immunostaining shows HER-2: IHC3+ or HER-2: IHC2+/FISH+

CI, confidence interval; EBV, Epstein-Barr virus; FISH, fluorescent in situ hybridization; HER-2, human epidermal growth factor receptor-2; *H. pylori, Helicobacter pylori*; IHC, immunohistochemistry; MSI-H, microsatellite instability-high; PD-L1, programmed death ligand-1

HER-2, human epidermal growth factor receptor-2; PD-L1, programmed death ligand-1.

### Association of PD-L1 expression with biomarkers

An association was observed between MSI-H tumors and PD-L1 protein expression, as a greater proportion of patients with MSI-H tumors (83.3% [20/24]) were PD-L1 positive than those with MSI-low/stable tumors (60.8% [216/355]; *p* = .0297). Similarly, an association was observed between history of *H. pylori* infection and PD-L1 expression, as a greater proportion of patients with a history of *H. pylori* infection (83.0% [44/53]) were PD-L1 positive compared with those without a history of *H*. *pylori* infection (54.7% [29/53]; *p* = .0030). However, no association was observed between PD-L1 expression and EBV-positive and HER-2–positive tumors (Table 2).

### Association of PD-L1 expression with H. pylori infection

An association was observed between history of *H. pylori* infection and PD-L1 expression, with a greater proportion of patients with a history of *H. pylori* infection (83.0% [44/53]) being PD-L1 positive compared with those without a history of *H. pylori* infection (54.7% [29/53]; *p* = .0030) (Table 2). The PD-L1–positive rate was higher in *H. pylori*–positive cases vs negative cases, and the expression of PD-L1 was more common in the CPS range of 1–<10 among those with vs without a history of *H. pylori* infection (52.8% [28/53] vs 32.1% [17/53]), i.e., PD-L1 expression at a lower CPS range was associated with positive infiltrating lymphocytes.

### Prevalence of PD-L1 expression based on clinicopathological parameters

No association was observed between PD-L1 expression and clinicopathological characteristics (*p* > .05 for all comparisons; Table 3). However, a greater proportion of PD-L1–positive vs PD-L1–negative patients had mixed type (65.3% vs 34.7%) of tumors, followed by intestinal type (64.2% vs 35.8%), diffuse type (60% vs 40%), and unclassifiable type (53.8% vs 46.2%) (Table 3).

**Table 3.** Prevalence of PD-L1 expression based on clinicopathological characteristics

Category*	Patients, *N* (%)	PD-L1–positive patients, *n* (%)	PD-L1–negative patients, *n* (%)
	***N* = 389 (100)**	**241 (62.0)**	**148 (38.0)**
**Site of occurrence**			
Stomach	369 (94.9)	230 (62.3)	139 (37.7)
Gastroesophageal junction	20 (5.1)	11 (55.0)	9 (45.0)
**Gastric cancer stage**			
IIA	3 (0.8)	2 (66.7)	1 (33.3)
IIB	6 (1.5)	3 (50.0)	3 (50.0)
IIIA	8 (2.1)	5 (62.5)	3 (37.5)
IIIB	8 (2.1)	3 (37.5)	5 (62.5)
IIIC	7 (1.8)	3 (42.9)	4 (57.1)
IV	266 (68.4)	177 (66.5)	89 (33.5)
Undetermined	91 (23.4)	48 (52.7)	43 (47.3)
**Sampling method**			
Biopsy	316 (81.2)	200 (63.3)	116 (36.7)
Surgical resection	73 (18.8)	41 (56.2)	32 (43.8)
**Histologic type per the Laurenclassification**			
Intestinal	137 (35.2)	88 (64.2)	49 (35.8)
Diffuse	190 (48.8)	114 (60.0)	76 (40.0)
Mixed	49 (12.6)	32 (65.3)	17 (34.7)
Unclassifiable	13 (3.3)	7 (53.8)	6 (46.2)

PD-L1, programmed death ligand-1

*p > .05 for all comparisons

### PD-L1 staining in cells based on CPS cutoffs

**Figure 4.**
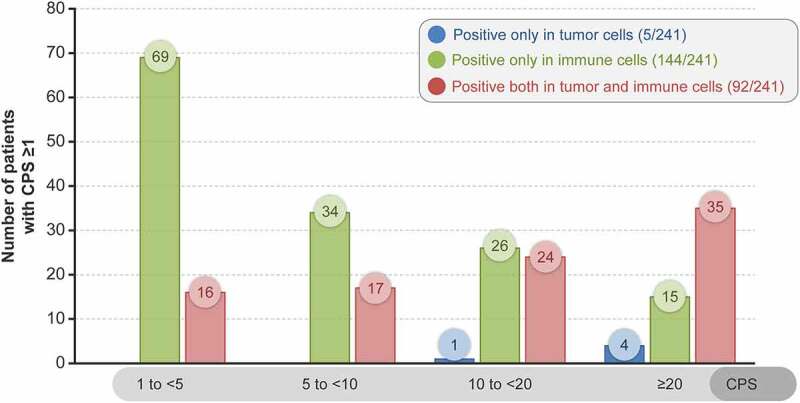
PD-L1-staining cells based on CPS cutoff

CPS, combined positive score; PD-L1, programmed death ligand-1.

Overall, tumor specimens were PD-L1 positive in immune cells only in 144/241 (59.8%) patients, in both immune cells and TCs in 92/241 (38.2%) patients, and only in TCs in 5/241 (2.1%) patients. Tumor specimens from 56.4% (136/241) of patients had a CPS ranging from 1 to <10 and those from 43.6% (105/241) of patients had a CPS ≥10 (Figure 4). A representative case of PD-L1 expression using IHC is presented in **Supplementary Figure 1**.

### Prevalence of biomarkers by PD-L1 cutoff

A higher proportion (66.7% [16/24]) of patients with MSI-H tumors demonstrated high PD-L1 expression with a CPS ≥10 than those with MSI-low/stable tumors (24.8% [88/355]; *p* < .0001). No associations were observed between other biomarkers (EBV, HER-2, or *H. pylori*) and high PD-L1 expression (CPS ≥10) (Table 4).

**Table 4.** Prevalence of biomarkers by CPS ≥10

Biomarker	Status	Total patients, *N*	Patients with CPS ≥10, *n* (%)	95% CI	Intergroup difference *p*-value
MSI-H	Positive	24	16 (66.7)	44.7, 84.4	<.0001
	Negative	355	88 (24.8)	20.4, 29.6	
EBV	Positive	13	6 (46.2)	19.2, 74.9	.1216
	Negative	376	99 (26.3)	21.9, 31.1	
HER-2	Positive	76	21 (27.6)	18.0, 39.1	1.000
	Negative	223	61 (27.4)	21.6, 33.7	
*H. pylori*	Positive	53	16 (30.2)	18.3, 44.3	.5091
	Negative	53	12 (22.6)	12.3, 36.2	

CI, confidence interval; CPS, combined positive score; EBV, Epstein-Barr virus; HER-2, human epidermal growth factor receptor-2; *H. pylori, Helicobacter pylori*; MSI-H, microsatellite instability-high

## Discussion

The PD-1/PD-L1 signaling cascade is an inhibitory factor in the cancer-immunity cycle.^28^ PD-L1 expression is thus assessed on both tumor-infiltrating immune cells (TIICs) and TCs for its predictive value. However, PD-L1 expression on TIICs might be more meaningful in terms of predictive value in specific tumors^18^ and response to immunotherapy.^8,13^ However, differences in molecular characteristics, including PD-L1 expression in the tumor microenvironment, might differ between types of cancers.^29–31^

This study evaluated the prevalence of PD-L1 expression, MSI-H status, and EBV positivity in Japanese patients with advanced gastric and GEJ adenocarcinoma. Although there have been several reports on the prevalence of PD-L1 in GC, their sample size was generally small, and real-world data for advanced cases are rarely reported.^32–35^ In this study, tumor specimens from 389 Japanese patients with advanced gastric or GEJ adenocarcinoma were collected consecutively between 2014 and 2019 and evaluated.

The prevalence of PD-L1 positivity (62%) in the real-world setting, assessed using the pharmDx IHC assay (CPS ≥1), is comparable to previously reported results from pembrolizumab clinical trials in GC.^26,27,35–38^ Among these PD-L1–positive patients (CPS ≥1, 62%), a similar proportion of patients had a CPS between 1 and <10 (56.4%) and ≥10 (43.6%), and this finding is in line with that of other studies in the Japanese population (27%–66%).^7,9,33^ However, PD-L1 expression was higher in the TIICs than in the TCs for lower CPS cutoffs (1 to <10), supporting the claim that expression on immune cells may be more meaningful as ignoring the expression in TIICs will lower the prevalence, especially for lower CPS cutoffs.

The prevalence of both MSI-H (6.3% by PCR) and EBV positivity (3.3% by ISH) in advanced GC was low in this study. However, of this small number of patients with MSI-H tumors (24/379; 6.3%), a higher proportion showed PD-L1 expression (83.3%), suggesting that MSI-H status is associated with PD-L1 positivity. Furthermore, higher PD-L1 expression (CPS ≥10) was observed in a higher proportion of patients with MSI-H tumors than those with MSI-low/stable tumors. While the proportion of MSI-H tumors was similar to that reported in studies from Japan and East Asia,^33,39,40^ it was lower than that reported in the TCGA project (stage I–III, 87.8% [259/295]; MSI, 21.2% [55/259] vs stage IV, 6.8% [20/295]; MSI, 10% [2/20]).^4,41^ These results support evidence from literature pertaining to(1) immune cell staining (CPS scoring) as a key characteristic in GC; (2) the overall, early-stage disease has a higher MSI-H prevalence than later-stage disease,^42^ and higher proportion of early-stage vs later-stage MSI-H gastric adenocarcinomas in patients with gastric/GEJ cancers screened in the clinical trial of pembrolizumab;^43^ (3) observations from studies reporting that the EBV-positive and MSI-H subtypes of GC are more likely to express PD-L1 when immune cells demonstrate a tumor-infiltrating pattern,^10−12^ indicating the involvement of the immune microenvironment in the development of GC;^8,13^ and (4) the role of MSI as a predictor for anti–PD-1/PD-L1 immunotherapy efficacy as demonstrated across tumor types.^29,30^ No relationship was observed when HER-2 status, EBV status, and histological subtype of GC per the Lauren classification were stratified by PD-L1 expression status (Table 2, Table 3, **Supplementary Figure** **1**).

Retrospectively, we also observed that a history of *H. pylori* infection might be associated with PD-L1 expression, and this warrants further investigation as it might not reflect the current biological disease state. *H. pylori*, the most common cause of GC, predominantly in East Asia,^44,45^ also induces PD-L1 expression in the gastric epithelium, increasing the risk of developing GC.^46^ The positive/negative test results for *H. pylori* were available only in patients with the test date recorded in the medical chart. As the testing method is not clear (blood, breath, or biopsy), a positive result (a history of infection) is not always indicative of the presence of *H. pylori*. Similarly, a negative test result (no history of infection) cannot differentiate whether it is just a decrease in the quantity of antibodies or if the patient was uninfected. Because 99% of Japanese patients with GC have a history of *H. pylori* infection,^47^ most individuals who tested negative in the present study should ideally have a history of infection. Patients may test negative depending on the quantity of antibodies and the timing of infection.^48,49^ Therefore, an *H. pylori*–negative status is dependent on the diagnostic method and represents the phase of infection over time,^49^ as a positive case is considered to have active inflammation and a negative case is considered to have less inflammation. Thus, our results may only perceive differences in the expression of PD-L1 among the different stages of inflammation. In the future, we hope that the effects of *H. pylori* on the immune milieu will be further clarified by examining PD-L1 expression in the inflamed phase or by examining the expression of PD-L1 in uninfected cases.

In our study, PD-L1 expression is observed more frequently in MSI-H tumors than in MSI-low/stable tumors without any significant relation to histological subtype. Higher tumor PD-L1 expression was reported in most MSI-H GC using CPS compared with MSI-stable GC.^50^ In the CheckMate 649 study, 4% (18/473) and 3% (16/482) of patients with a PD-L1 CPS of ≥5 had MSI-H tumors in the nivolumab plus chemotherapy and chemotherapy alone groups, respectively,^51^ and some MSI-H patients may not express PD-L1 at high levels or may have a CPS of 1–5.^50,52^ However, we must note that differences exist in antibody IHC assays (22C3 and 28-8Ab), definitions and cutoffs of CPS, and methods for detecting MSI (IHC vs PCR).

A recent meta-analysis of randomized controlled trials has shown that patients with MSI-H GC should be considered as a specific population that is highly immunosensitive.^53^ Hence, immunotherapy could be an option in this subset of patients. Furthermore, a correlation between MSI-H tumors and immune checkpoint ligands such as PD-L1 has been indicated^54^ due to the increased number of neoantigens present in the MSI-H subtype, leading to the stimulation of PD-L1 through the secretion of interferon gamma by T lymphocytes.^55^

Additionally, an analysis of MSI status could help assess the tumor microenvironment and provide insights into the most appropriate treatment option.^39^ Therefore, patients with MSI-H tumors could benefit from an immunotherapeutic approach. In concordance with this hypothesis, an evaluation of the KEYNOTE-158 and KEYNOTE-164 studies showed robust antitumor activity of pembrolizumab in heavily pretreated patients with MSI-H cancers.^56,57^ Pembrolizumab is approved for the treatment of MSI-H solid tumors after failure of current standard therapy.^58^ It is known that the EBV subtype accounts for 8.8% of all GC subtypes and is associated with a better prognosis.^41,59^ Interestingly, the prevalence of EBV (3.3%) was low in our study compared with that in studies in Asia and Latin America (7.7%)^60^ and Japan (approximately 5.3%^61^ and 5.1%^32^). This could be attributable to the difference in the ISH protocol used for this analysis. The EBV subtype of GC usually has specific histological and clinical features such as intra- or peritumoral lymphocytic infiltration (carcinoma with lymphoid stroma).^60–62^ None of the tumor specimens obtained in our study were of the carcinoma with lymphoid stroma type. This unusual histological finding could be due to a bias in sampling, since only advanced lesions were collected for this study. Additional research is therefore required to further evaluate the biological behavior of EBV-positive adenocarcinoma without lymphoid stroma.

No association between PD-L1 expression and EBV-infected tumor type was observed, possibly due to the small cohort of patients with EBV-positive tumors. However, previous studies have demonstrated an overexpression of PD-L1 in EBV-positive GC.^55,63^ Indeed, a combined analysis of 4 studies involving 1307 Japanese patients indicated a significant association between EBV positivity and PD-L1 expression (*p* < .0001).^10^ Similar findings have also been reported in studies from Japan.^11,32^

Our study did not show an association between HER-2 and PD-L1 expression. To date, contrasting results have been reported indicating PD-L1 expression in HER-2–negative^64^ and HER-2–positive tumors,^65^ and further research is therefore warranted to confirm this association. When HER-2 status was characterized by histological subtype, HER-2 positivity was most common in the intestinal subtype of GC, which is in line with that reported in previous studies.^66,67^ Interestingly, 91.7% of diffuse HER-2–positive tumors were PD-L1 positive.

Further, we did not observe any association between PD-L1 expression using CPS and patient clinicopathological characteristics/parameters. However, a recent study by Kawazoe and colleagues^33^ reported an association between PD-L1 expression and clinicopathological parameters (TCs: mismatch repair deficient, *PIK3CA* mutation, and *KRAS* mutation; immune cells: EBV positivity and lymph node metastasis) using the IHC 22C3 pharmDx kit in metastatic GC, but IC and TC were scored separately in the study. Other characteristics such as tumor size and lymph node status have been associated with PD-L1 positivity.^68^ Overall, 47.3% (114/241) of all PD-L1–positive specimens were of the diffuse cancer subtype; this finding is in line with a retrospective study that reported an association between the diffuse type GC and PD-L1 positivity, indicating that histological characteristics should be considered when selecting patients who may benefit from anti–PD-L1 therapy.^69^

To our knowledge, this is the first real-world study that used the CPS method to evaluate PD-L1 expression in Japanese patients with gastric and GEJ adenocarcinoma. A number of studies have stressed the importance of evaluating PD-L1 expression not only on TCs but also on TIICs.^8,9,13,70,71^ PD-L1 expression on TIICs has a stronger relationship with the cancer immune response than PD-L1 expression only on TCs.^8^ Most responders to pembrolizumab were identified when tumor and immune cell PD-L1 expression were combined,^18^ making CPS a more sensitive measure/scoring system compared with tumor proportion score (TPS).^17–19^ However, there are certain limitations of this study. This was a retrospective analysis that used archived tissue. In addition, since this was a multisite study, selection bias between the study sites may have existed.

## Conclusion

The prevalence of PD-L1 positivity (62%) in the real-world setting using the pharmDx IHC assay (CPS ≥1) was comparable to previously reported results from pembrolizumab clinical trials and other exploratory studies in GC.^26,27,33,35–38,72^ PD-L1 staining was observed more frequently in TIICs than in TCs for a lower CPS cutoff. The rate of MSI-H and EBV-positive tumors in advanced GC was 6.3% and 3.3%, respectively. We observed that the MSI-H status is associated with PD-L1 positivity, suggesting high number of tumor neoantigens. We also observed that a history of *H. pylori* infection might be associated with PD-L1 expression; however, further investigation is warranted to confirm this finding.

## Supplementary Material

Supplemental Material
