# Supplementary material for: Clinicopathological features of PD-L1 protein expression, EBV positivity, and MSI status in patients with advanced gastric and esophagogastric junction adenocarcinoma in Japan
Source: Cancer Biol Ther. 2022 Feb 27;23(1):191–200. doi: 10.1080/15384047.2022.2038002 (PMC8890430; doi:10.1080/15384047.2022.2038002)
Supplement: Supplemental Material [file KCBT_A_2038002_SM3383.docx]

**Supplementary Figure 1.** A representative case of PD-L1 expression using IHC

CPS, combined positive score; EBV, Epstein-Barr virus; GC, gastric cancer; HE, hematoxylin and eosin; HER-2, human epidermal growth factor receptor-2; *H. pylori*, *Helicobacter pylori*; IHC, immunohistochemistry; MSS, microsatellite stable; MSI-H, microsatellite instability-high; PD-L1, programmed death ligand-1
